# Design and Performance of a Composite Grating-Coupled Surface Plasmon Resonance Trace Liquid Concentration Sensor

**DOI:** 10.3390/s19245502

**Published:** 2019-12-12

**Authors:** Wenchao Li, Zhiquan Li, Jiahuan He, Liyang Chu

**Affiliations:** 1 School of Control Engineering, Northeastern University at Qinhuangdao, Qinhuangdao 066004, China; liwenchao@neuq.edu.cn; 2 Institute of Electrical Engineering, Yanshan University, Qinhuangdao 066004, China; 13476275587@163.com (J.H.); 13941737414@163.com (L.C.)

**Keywords:** surface plasmon, resonance, grating, Au/ITO, sensor, coupling

## Abstract

In this paper, a grating-coupled surface plasmon resonance concentration sensor employing a gold and indium tin oxide (Au/ITO) nanoparticle composite instead of metal is proposed. The structure and material parameters of the sensor are discussed and analyzed. Taking the ethylene glycol concentration as an example, the influence of the nanocomposite on the wave vector matching, the influence of the refractive index of the medium to be tested and the influence of the concentration on the refractive index were analyzed in detail. The experimental results show that when the sensor is used for the measurement of ethylene glycol concentration, the correlation coefficient between the concentration and the refractive index is as high as 0.999995. The fitting curve and data correlation are good, and the sensitivity has a good linear relationship with the sensitivity. Therefore, the sensor structure proposed in this paper can be used to accurately measure the trace concentration of liquid, and its sensing mode has certain reference value for the measurement of general trace fluid concentration.

## 1. Introduction

Surface plasmon resonance (SPR) sensors take advantage of the wavelength shift of the light reflected by SPR due to differences in the refractive index of the test medium [1,2,3,4]. SPR amplifies and transforms weak signals into measurable optical signals and obtains the information of the sample through spectral and numerical analyses. Stem and Farre found that SPR is caused by energy changes, analyzed the related resonance conditions, and summarized the dispersion relations of SPR. In 1968, Otto proposed that, when the incident angle of light is greater than a critical angle, total reflection occurs at the metal–medium interface and can produce plasmon resonance excitation on the interface [5]. The Otto structure is proposed based on this phenomenon. In 1970, Restchmann modified the Otto model and proposed a Kretschmann-type structure that stimulated SPR [6]. In 2010, L. Wu et al. proposed a SPR-based graphene biosensor, which consists of a graphene sheet coated above a gold thin film, the attenuated total reflection (ATR) method is used to detect the refractive index change near the sensor surface, and the results show that the proposed SPR biosensor is more sensitive than the conventional gold thin film SPR biosensor [7]. In 2019, Y. Xu et al reviewed six major categories of optical refractive index sensors using plasmonic and photonic structures, which contained metal-based propagating plasmonic eigenwave structures, metal-based localized plasmonic eigenmode structures, dielectric-based propagating photonic eigenwave structures, dielectric-based localized photonic eigenmode structures, advanced hybrid structures and 2D material integrated structures [8]. Then, they combined the two-dimensional Ti_3_C_2_Tx MXene and transition metal dichalcogenides to enhance the sensitivity of SPR, which could achieve a high sensitivity of 198°/RIU (refractive index unit) with a sensitivity enhancement of 41.43% in aqueous solutions (refractive index ∼1.33) with the employment of monolayer Ti_3_C_2_Tx MXene and five layers of WS_2_ at a 633 nm excitation wavelength [9].

SPR sensing technology has been widely used in food safety, biochemical research, environmental monitoring, and many other fields [10,11,12]. SPR sensors have a large number of advantages, such as label-free detection, real-time monitoring, high sensitivity, small sample requirements, a wide range of application and non-invasive analysis, and therefore it has quickly become a popular study topic in optoelectronics research [13,14]. Recent work demonstrates that, under special circumstances, for example, in detecting the concentration of pesticide residues in food, measuring the interaction of gas and liquid components, and the biomass of signal molecules on the biofilm of the medium to be measured, and so on, SPR sensors show poor performance [15,16]. Thus, in this work, we propose a new design of SPR sensors for the detection of trace gas and liquid concentrations.

## 2. Sensor Structure Design Principle and Preparation Method

### 2.1. Sensor Structure Design

**Figure 1:**
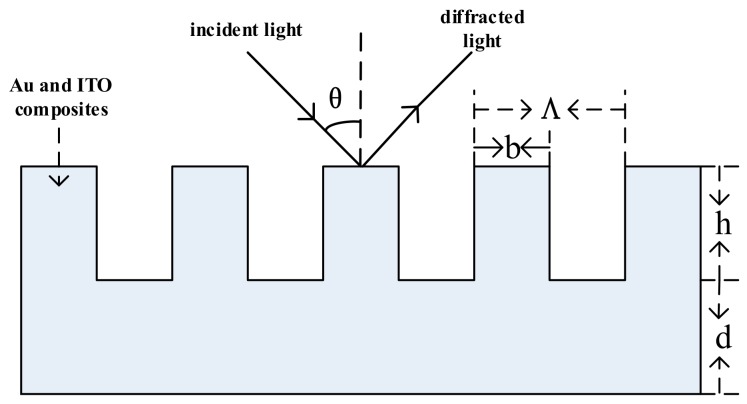
The composite grating coupling surface plasmon resonance (SPR) sensor design structure diagram.

In this paper, the structure of a sensor using a gold and indium tin oxide (Au/ITO) nanocomposite is presented. As shown in Figure 1, among the grating structures: grating depth h = 35 nm; grating width b = 180 nm; grating period Λ = 1000 nm; nanocomposite grating substrate thickness d = 285 nm; duty cycle Ω = 0.82. The sensor production process is: pyrex glass deposition of 40 nm armor grease polymethyl methacrylate (PMMA) is executed to form a corrosion-resistant layer, on the corrosion layer with uniform glue machine spin 300 nm Au/ITO hybrid composites of colloid particles. After drying in pure nitrogen, a sensing membrane that is sensitive to the solution being tested and capable of identifying the substance being tested was modified. After washing away unbound material, the substance was placed in a 4 °C refrigerator for 12 h. Plasmon dry etching with 300 nm Au/ITO nanocomposites was performed up to a depth of 35 nm, as shown in Figure 1. Raster layer was 285 nm subwavelength grating structure, as a sensor is sensitive to the chip. Among nano-structured particles, Au nano-structured particles have good characteristics such as large specific surface area, strong corrosion resistance, oxidation resistance and chemical inertness, and good stability. ITO is a low refractive-index medium. This paper uses Au/ITO nanocomposite materials instead of traditional metal materials. Under a certain fixed angle of polarized light, the dielectric constant of the nanocomposite materials is controlled by adjusting the volume ratio of Au to ITO. The SPR resonance output of this nanocomposite layer has two resonance peaks, namely, the long-range surface plasmon resonance peak (peak 1) and the short-range surface plasmon resonance peak (peak 2). The wavelength of peak 1 produces a corresponding drift with changes in the refractive index of the test medium. The wavelength of peak 2 does not drift with changes in the refractive index of the test medium and only depends on the wavelength of the incident light, the incident angle, and the dielectric constant of the composite material layer. Au/ITO nanocomposite-based SPR sensors not only have high sensitivity and precision but also provide a peak wavelength that does not change with the refractive index of the test medium and short reference resonance peak drift. Moreover, the reference peak can reduce the effect of the incident light angle drifts on the measured results.

In this work, we conducted theoretical analysis and simulation experiments using the designed SPR sensor. The incident wavelength, the refractive index of the test medium, and the structural parameters of the sensing system are analyzed in terms of wavelength modulation and a fixed incident angle.

### 2.2. Working Principle of the Sensor 

**Figure 2:**
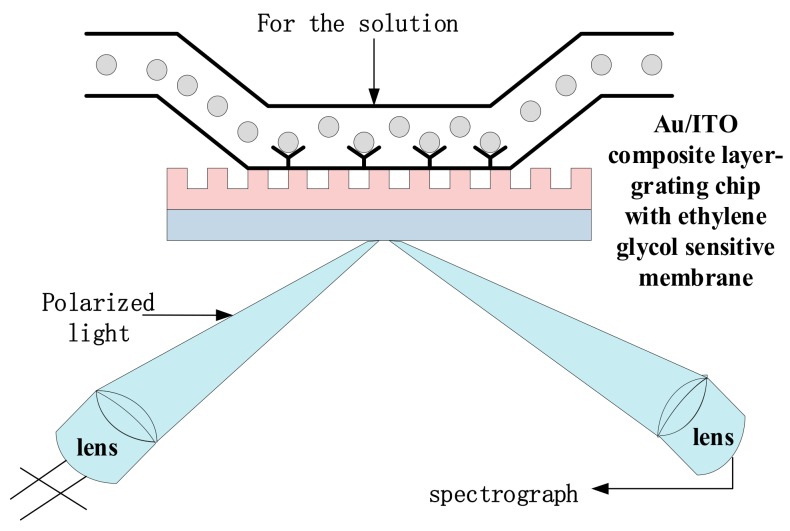
The schematic diagram of SPR biosensor.

As shown in Figure 2, the corresponding sensing membrane is fixed to the grating surface, and the solution under test flows through the grating chip. The analyte and sensor membrane have binding specificity and form a complex under certain conditions. The analyte is not filled in the grating gaps, and just a layer of analyte is tiled on the surface of the grating for binding to the sample. The process takes only a few seconds to complete. The combination of antigen and antibody can cause mass changes on the surface of the sensor. As the refractive index of the diffracted light is proportional to the change in mass, the refractive index changes accordingly. The relationship between the incident wavelength and the refractive index of the medium is presented by the sensor mechanism. When P-polarized light enters the diffraction grating through the flow channel, the surface biomolecule recognition element is adsorbed on the grating, which can capture specific analyte molecules, causing the refractive index of the surface on the grating to change and the resonance angle in the reflection spectrum to shift. The functional relationship between the angular offset and the properties of the measured sample is used to analyze the characteristics of the sample.

The fact that biosensor chips can be reused is good proof that the chips have practical value. Since the binding of antigenic antibody is the non-covalent bonding on the molecular surface, the reaction of antigenic antibody is reversible. An extremely high or low pH and enhanced ionic strength can destroy electrostatic attractions and dissociate the complex formed. The chip retains their original biological activities after dissociation. In this computational study, 0.1 mol/L HCl was used as the eluent, the reaction-generating substance and the remaining substance on the sensing membrane can be effectively eluted, and the sensor chip can be modified multiple times for repeated use.

## 3. Influence of Materials and Measured Medium on Sensor Performance

### 3.1. Effect of Nanocomposites on Sensor Performance

**Figure 3:**
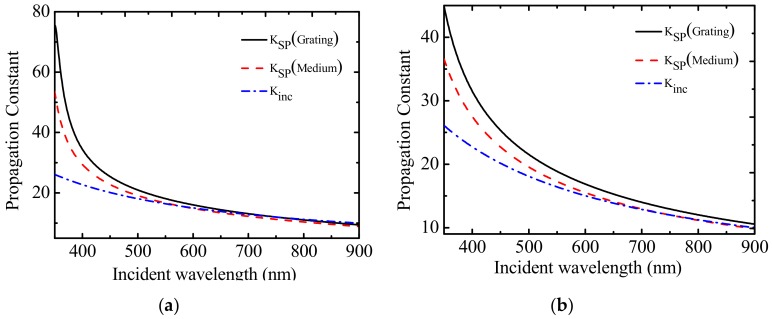
The influence of dielectric constant of metal layer on wave vector matching. (**a**) Dielectric constant characteristic waveform of gold and indium tin oxide (Au/ITO) nanocomposites. (**b**) Dielectric constant characteristic waveform of Au.

Figure 3 shows the change of the propagation constant of the surface plasmon wave and the incident light with the incident wavelength at the interface of the dielectric layer to be measured. The influence of Au/ITO composite and gold on the surface plasmon wave vector of the sensor at the interface of the grating–composite layer (metal layer) and the interface of the composite layer (metal layer)–test medium layer was analyzed; and the influence of Au/ITO composite and gold on the matching of the surface plasmon wave vector with the wave vector of incident light is also analyzed.

As shown in Figure 3a, the Kinc curve of the propagation constant of incident light and the two curves of the propagation constant of the plasmon wave Ksp show an intersection point, that is, the propagation constant of incident light at different wavelengths is equal to the propagation constant of the plasmon wave at the two interfaces. Therefore, resonance can be excited at the interface between the composite layer of the diffraction grating and the dielectric layer to be measured. Two resonance peaks are present in the reflection spectrum, one of which is related to the refractive index of the medium to be measured; the other peak is only related to the wavelength of the incident light, the incident angle, and the dielectric constant of the composite layer. When Au/ITO nanocomposites replace the metal layer, the wave vector of the plasmon wave at the interface of the metal layer changes due to the characteristic of the dielectric constant of the composite material, and the sensor’s reflection spectrum shows two resonance peaks. As shown in Figure 3b, when the metal material is gold, the curve of the propagation constant Kinc of incident light and the curve of the propagation constant Ksp of the plasma wave at the interface between the composite material layer and the dielectric layer to be measured have only one intersection. This means that the wave vector of incident light can only excite the surface plasmon resonance of the interface between the gold layer and the dielectric layer to be measured. Thus, the reflection spectrum has only one resonance peak related to the refractive index of the dielectric to be measured.

### 3.2. Influence of Refractive Index of the Dielectric to be Tested on Sensor Performance

**Figure 4:**
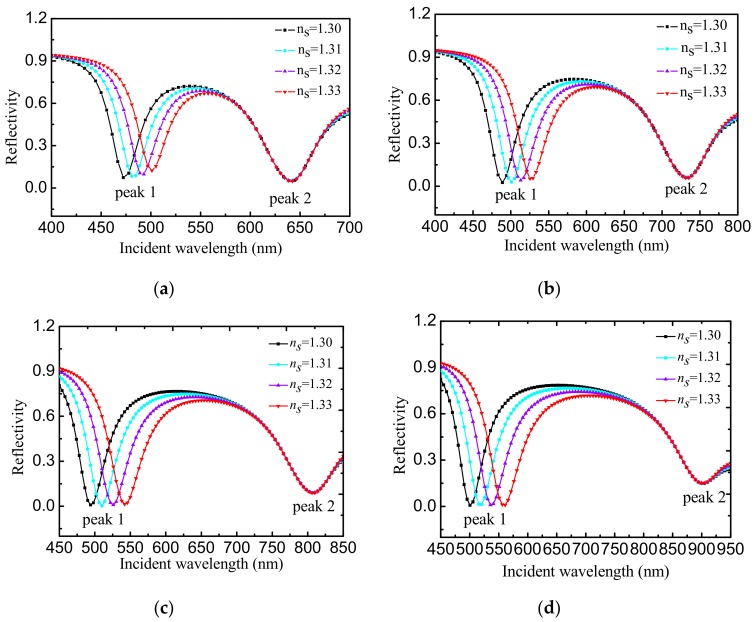
Reflectance spectra of different refractive indices of the medium to be tested (nanocomposite layer thickness d = 40 nm). (**a**) volume fraction f = 0.55, (**b**) volume fraction f = 0.65, (**c**) volume fraction f = 0.75, and (**d**) volume fraction f = 0.85.

Figure 4 shows the change of the reflection spectrum of Au/ITO nanocomposite sensor with the refractive index of the medium to be measured. The refractive index of the medium being tested ranges from 1.30 to 1.33. As the refractive index of the medium under test increases, when the volume fraction of glycol solution is at f = 0.55, the resonance wavelength of reflectivity dip 1 changes from 472 nm to 501 nm, and the resonance peak 2 changes from 639 nm to 640 nm. When f = 0.65, the resonance wavelength of reflectivity dip 1 changes from 489 nm to 525 nm, and the resonance wavelength of reflectivity dip 2 remains unchanged at 733 nm, which is determined by the properties of Au/ITO nanocomposite. If f is the volume ratio of Au and ITO in the composite, then when f = 0.75, the resonance wavelength of reflectivity dip 1 changes from 494 nm to 544 nm, and the resonance wavelength of reflectivity dip 2 changes from 805 nm to 806 nm. When f = 0.85, the resonance wavelength of reflectivity dip 1 changes from 501 nm to 556 nm, and the resonance wavelength of reflectivity dip 2 changes from 899 nm to 900 nm. It can be seen that under the four values of f, the resonance wavelength of SPR curve reflectivity dip 2 changes with the refractive index by a maximum of 1 nm, with a small change. In addition, the larger f is, the larger the resonance wavelength of reflectivity dip 1 becomes with the refractive index of the medium under test, and the sensitivity of the sensor increases accordingly.

## 4. Trace Concentration Measurement and Experimental Study

### 4.1. Effect of Solution Concentration on Refractive Index

In previous studies, the refractive index of a liquid at constant temperature has been proven to depend on the concentration of the liquid via a quantitative relationship. Therefore, liquid solutions with different refractive indices can be obtained by selecting solutions with different volume fractions during the experiment. The relationship between the refraction index *n* of the mixed solution and the volume *V_1_* of the solute, the volume *V_2_* of the solvent, the refraction index *n_1_* of the solute, and the refraction index *n_2_* of the solvent before mixing satisfies the following logarithmic mixing rule:

(1) $$\mathrm{lg}n=\frac{V_{1}}{V_{1}+V_{2}}\mathrm{lg}n_{1}+\frac{V_{2}}{V_{1}+V_{2}}\mathrm{lg}n_{2}$$

The volume fraction and the quality fraction of the relationship into (1) are as follows:

(2) $$w=\frac{\rho_{1}\ln n_{2}-\rho_{1}\ln n}{\left(\rho_{2}-\rho_{1}\right)\ln n-\left(\rho_{2}\ln n_{1}-\rho_{1}\ln n_{2}\right)}$$

where $\rho_{1}$ and $\rho_{2}$ are the density of the solute and solvent, respectively, n is the solution quality, and w is the quality fraction.

According to Equations (1) and (2), as long as the wavelength corresponding to the SPR sensor resonance peak is analyzed, the solution mass fraction *w* can be deduced.

At room temperature, the index of refraction of water is $n_{1}=1.3333$ and the index of refraction of ethylene glycol is $n_{2}=1.4329$. On the basis of Equation (1) and Equation (2), the sensor prototype made by the project team was used to measure the concentration of ethylene glycol solution. The data in Table 1 show the refractive index of glycol at various volume concentrations.

**Figure 5:**
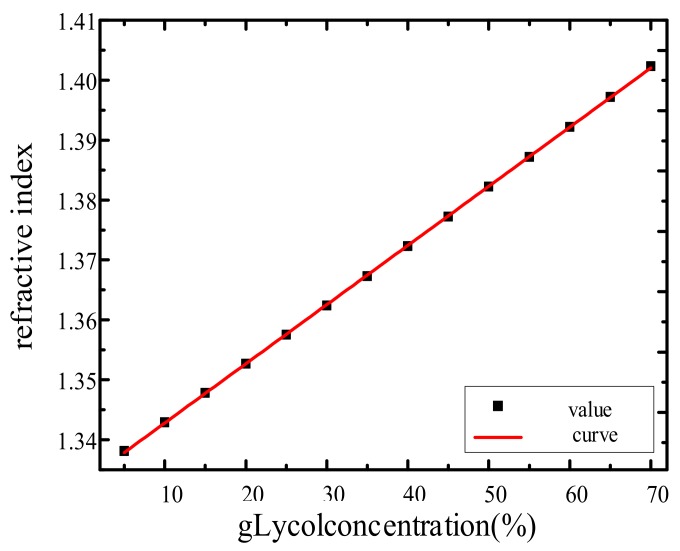
Relation between solution concentration and refractive index.

Using the data in Table 1, the fitting curve of the relationship between glycol concentration and refractive index was obtained. As shown in Figure 5, the refractive index equation is n = 1.33293 + 0.09872c, and the correlation coefficient of the fitting equation is 0.999995 which is extremely closed to 1. Therefore, we can see that the fitting effect is very good, which means that the concentration and refractive index have a strong linear relationship.

### 4.2. Relationship between the Resonance Wavelength and Solution Concentration

**Figure 6:**
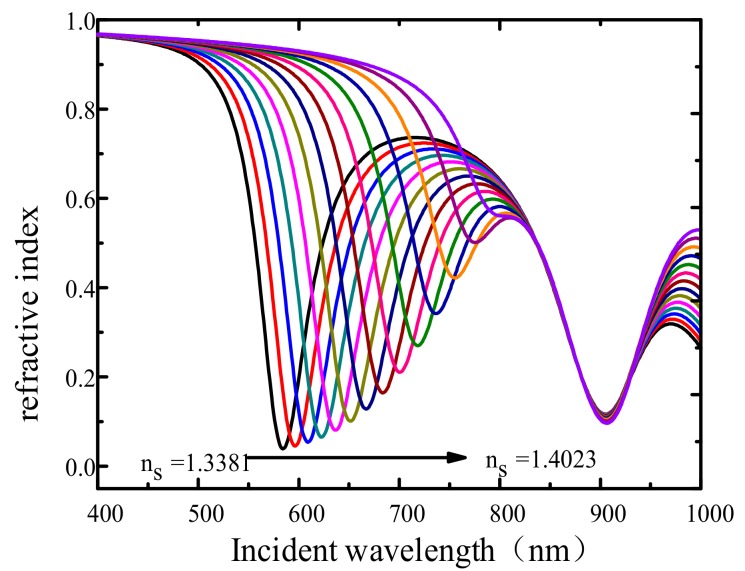
The relation between solution concentration and refractive index.

As the refractive index of ethylene glycol increases, the resonance depth of resonance peak 1 in the reflection spectrum decreases. When the volume concentration of ethylene glycol is 70% and the refraction index of the solution is 1.4023, no obvious resonance peak is observed in the reflection spectrum, as shown in Figure 6. This finding is due to the solution refractive index being close to the grating refractive index (about 1.45), which leads to an evanescent wave that cannot be coupled with the plasmon wave on the metal surface. Thus, no SPR effect is generated.

When determining the relation between the solution refractive index and the wavelength of reflectivity dip 1, a large error occurs during linear fitting. Thus, quadratic polynomial non-linear fitting is adopted to obtain the relation between the wavelength and the refractive index, as shown in Equation (3):

(3) $$\lambda =21,195.588-33,254.68n+13,340.684n^{2}$$

**Figure 7:**
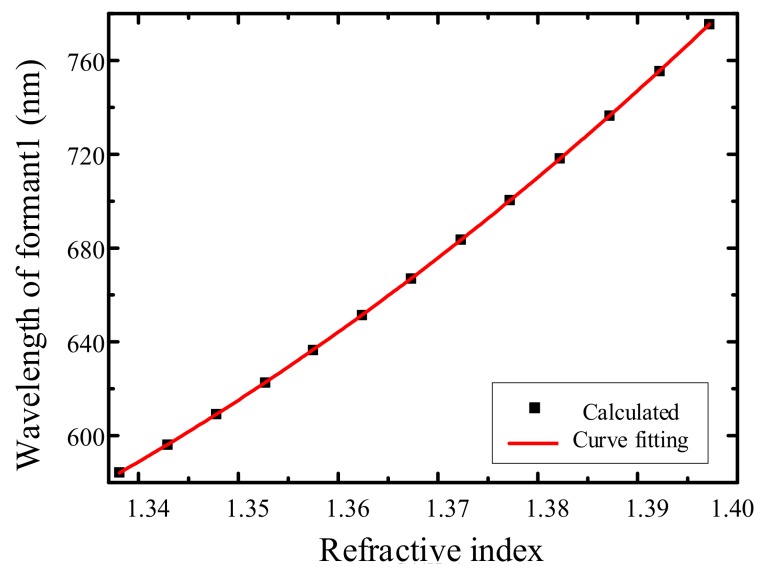
The relation between refractive index and resonance wavelength.

The correlation coefficient is 1, and the fitting curve is shown in Figure 7.

The correlation between the fitted curve and the measured value is R = 1, and the fitting degree is high. Equation n = 1.33293 + 0.09872c, which is the relation between the fitted refractive index and the solution volume concentration, is substituted in the equation between the refractive index and the wavelength of reflectivity dip 1. Thus, the relation between the wavelength of Equation (4) and concentration *c* is obtained as follows:

(4) $$\lambda =k_{1}-k_{2}(k_{3}+k_{4}c)+k_{5}{\left(k_{3}+k_{4}c\right)}^{2}(c>0)$$

Equation (5) can be achieved by solving Equation (4):

(5) $$c=-k_{6}+\sqrt{-k_{7}+k_{8}\lambda }$$

where the values of $k_{1}$, $k_{2}$, $k_{3}$, $k_{4}$, $k_{5}$, $k_{6}$, $k_{7}$, $k_{8}$ are shown in Table 2.

The volume concentration of glycol can be obtained by identifying the resonance wavelength of the SPR sensor. Taking the derivative of the concentration *c* with respect to the wavelength, the sensitivity of the sensor can be obtained as follows:

(6) $$S_{\lambda }=260.03c+228.02(c>0)$$

Table 3 shows that, when the incident angles are 80° and 80.5°, the two reflectivity dips vary with the volume concentration. When the incident angle increases to 0.5°, one resonance peak drifts to the left by at least 5 nm, and reflectivity dip 2 drifts to the left by 1 nm. During measurement, traditional sensors are unable to detect the influence of the incident angle easily. However, the self-reference sensor can express the drift of the incident wavelength through another reflectivity dip drift. Hereby, the large drift in reflectivity dip 2 indicates that the incident wavelength has changed. At this point, the incident wavelength must be adjusted to return reflectivity dip 2 to its original position. After the sensor is calibrated, measurement can be continued. Compared with the traditional SPR sensor, as shown in reference [7], the proposed sensor weakens the influence of the incident wavelength shift on the measurement results and increases their reliability.

In this computational study, an SPR sensor prototype was used to measure the concentration of ethylene glycol solution, and the relationship between the concentration and wavelength at resonance peak 1 was obtained. If reflectivity dip 2 is taken as the reference point, when reflectivity dip 2 changes, the incident angle must be readjusted and measured once more.

## 5. Conclusions

In this paper, an Au/ITO nanocomposite SPR sensor is designed, and the effect of the dielectric constant of the metal layer on the resonant peak of the sensor is analyzed. Owing to ITO doping, the wave vector of the incident light can match the wave vector of the surface plasmon wave at the interface of the grating and the nanomaterial layer dielectric layer to be measured to generate a resonance mode. Reflectivity dip 1 appears at the interface between the nanomaterial layer and the dielectric layer to be measured, which is related to the refractive index of the dielectric. Reflectivity dip 2 occurs at the diffraction grating–nanomaterial interface and is independent of the refractive index of the medium being tested. This paper also discusses the characteristics of the sensor and carries out simulation and laboratory experiments. The sensor is used to measure the concentration of ethylene glycol solution, and the influence of temperature and concentration on the sensor is discussed. As the concentration is not linearly related to the wavelength at reflectivity dip 1, the sensitivity of the sensor is not constant but linearly related to the concentration. The theoretical research and simulation experiments carried out in this paper can be applied to the development and application of SPR biological and chemical sensors.

## Figures and Tables

**Table 1:** The change of refractive index of ethylene glycol solution with concentration.

Concentration/%	Refractive Index	Concentration/%	Refractive Index
5	1.3381	40	1.3723
10	1.3429	45	1.3772
15	1.3478	50	1.3822
20	1.3527	55	1.3872
15	1.3575	60	1.3922
30	1.3624	65	1.3972
35	1.3673	70	1.4023

**Table 2:** The values of $k_{1}$, $k_{2}$, $k_{3}$, $k_{4}$, $k_{5}$, $k_{6}$, $k_{7}$, $k_{8}$.

$k_{1}$	$k_{2}$	$k_{3}$	$k_{4}$	$k_{5}$	$k_{6}$	$k_{7}$	$k_{8}$
21195.588	33254.68	1.33293	0.09872	13340.684	0.87689	3.62947	0.00769

**Table 3:** Changes of two resonance peaks with volume concentration at different incident wavelengths.

Concentration (%)	Incident Angle α = 80°		Incident Angle α = 80.5°	
	Reflectivity Dip 1 (nm)	Reflectivity Dip 2 (nm)	Reflectivity Dip 1 (nm)	Reflectivity Dip 2 (nm)
5	584	905	580	904
10	596	905	591	904
15	609	905	604	904
20	623	905	617	904
25	636	905	631	904
30	651	905	645	904
35	667	905	660	904
40	684	905	677	904
45	700	905	693	904
50	718	905	711	904
55	737	906	729	905
60	755	906	748	905
65	775	906	767	905
